# Genetically Determined Response to Artemisinin Treatment in Western Kenyan *Plasmodium falciparum* Parasites

**DOI:** 10.1371/journal.pone.0162524

**Published:** 2016-09-09

**Authors:** Lorna J. Chebon, Bidii S. Ngalah, Luicer A. Ingasia, Dennis W. Juma, Peninah Muiruri, Jelagat Cheruiyot, Benjamin Opot, Emmanuel Mbuba, Mabel Imbuga, Hoseah M. Akala, Wallace Bulimo, Ben Andagalu, Edwin Kamau

**Affiliations:** 1 Department of Emerging and Infectious Diseases, United States Army Medical Research Directorate-Kenya, Kenya Medical Research Institute (KEMRI)/Walter Reed Project, Kisumu, Kenya; 2 Institute of Tropical Medicine and Infectious Diseases, College of Health Sciences, Jomo Kenyatta University of Agriculture and Technology, Nairobi, Kenya; 3 Ifakara Health Institute, Bagamoyo, Tanzania; Institut national de la santé et de la recherche médicale - Institut Cochin, FRANCE

## Abstract

Genetically determined artemisinin resistance in *Plasmodium falciparum* has been described in Southeast Asia. The relevance of recently described Kelch 13-propeller mutations for artemisinin resistance in Sub-Saharan Africa parasites is still unknown. Southeast Asia parasites have low genetic diversity compared to Sub-Saharan Africa, where parasites are highly genetically diverse. This study attempted to elucidate whether genetics provides a basis for discovering molecular markers in response to artemisinin drug treatment in *P*. *falciparum* in Kenya. The genetic diversity of parasites collected pre- and post- introduction of artemisinin combination therapy (ACT) in western Kenya was determined. A panel of 12 microsatellites and 91 single nucleotide polymorphisms (SNPs) distributed across the *P*. *falciparum* genome were genotyped. Parasite clearance rates were obtained for the post-ACT parasites. The 12 microsatellites were highly polymorphic with post-ACT parasites being significantly more diverse compared to pre-ACT (p < 0.0001). The median clearance half-life was 2.55 hours for the post-ACT parasites. Based on SNP analysis, 15 of 90 post-ACT parasites were single-clone infections. Analysis revealed 3 SNPs that might have some causal association with parasite clearance rates. Further, genetic analysis using Bayesian tree revealed parasites with similar clearance phenotypes were more closely genetically related. With further studies, SNPs described here and genetically determined response to artemisinin treatment might be useful in tracking artemisinin resistance in Kenya.

## Introduction

Artemisinin combination therapies (ACTs) remain the mainstay treatment of uncomplicated malaria in most malaria endemic regions despite confirmed resistance in Southeast Asia (SEA) [1], which is characterized by slow parasite clearance rates [2,3]. Sensitive *Plasmodium falciparum* parasites are cleared within 48 hours in 95% of the patients after treatment with artemisinin when assessed using blood smears [4]. Presence of parasites at 72 hours is a predictor of subsequent treatment failure [5]. A recent study which mapped the geographic extent of resistance in SEA and Africa found median parasite clearance half-lives ranging from 1.9 hours in the Democratic Republic of Congo (DRC) to 7.0 hours at the Thailand—Cambodia border [6]. Although parasites occasionally had clearance half-life > 5 hours in DRC and Nigeria, in line with previous studies, ACTs remain highly effective in Africa with fast clearance half-lives [7–9]. Unlike parasites from SEA that showed strong association of clearance half-lives with point mutations in the recently described K13-propeller region [10], there was no association of K13 polymorphisms with parasite clearance half-lives in African parasites [6]. Indeed, the recently described mutations in the K13-propeller region in SEA parasites have not been found in African parasites [6, 11–13].

Genetically determined artemisinin resistance in *P*. *falciparum* has been described in SEA [14–16]. In western Cambodia, parasite clearance rates have been shown to be a heritable parasite-encoded trait where patients with similar parasite clearance rates are infected with clonally identical (CI) parasites [14]. The proportion of genetically determined artemisinin resistance has increased substantially in SEA, with parasite clearance attributable to parasite genetics increasing from 30% between 2001 and 2004, to 66% between 2007 and 2010 [14]. Further, a recent study discovered several distinct but apparently sympatric parasite subpopulations with extremely high levels of genetic differentiation in Cambodia [15]. Three subpopulations associated with clinical resistance to artemisinin had skewed allele frequency spectra and high levels of haplotype homozygosity, which is indicative of founder effects and recent population expansion. Interestingly, the K13-propeller alleles were shown to be strongly associated with parasite subpopulations [10].

*Plasmodium falciparum* population structure is correlated with the intensity of malaria transmission. Regions of low transmission intensity such as SEA results in higher rates of inbreeding and greater population structure unlike regions of high transmission intensity such as sub-Saharan African (SSA) countries [16,17]. Studies have shown that resistance to several antimalarial drugs originated in western Cambodia where parasite populations are highly structured and then spread to the rest of the world [18]. Parasite populations in endemic areas of SSA differ both genotypically and phenotypically in terms of virulence, drug resistance and transmissibility [19]. It is yet to be understood whether parasites in regions of high transmission intensity which are less structured carry parasite populations or subpopulations that are genetically similar with heritable traits such as artemisinin resistance.

Studies have shown that the number of genotypes per person directly correlates with the transmission intensity [20, 21]. Genome-wide association studies (GWAS) are carried out to determine the presence of genetic markers influencing a phenotypic trait throughout the parasite genome and can be used to identify genomic region(s) or parasite gene(s) that is responsible for causing resistance to antimalarial drugs [22]. The discovery that none of the previously described important K13-propeller mutations associated with artemisinin resistance found in SEA exist in African parasite population [6, 11–13] was insightful. However, it remains unknown whether parasite genetics can provide a basis for discovering molecular markers for artemisinin resistance in *P*. *falciparum* from SSA given the parasite genetic plasticity. To address this question, we analyzed the genetics and population structure of parasite from western Kenya, a region of high malaria transmission. Samples used in this study were collected before (pre) and after (post) the introduction of Artemether-Lumefantrine (AL) as the first-line ACT in Kenya. The post-ACT parasites were from an efficacy trial where parasite clearance rates were obtained.

## Results

### Microsatellite analysis

Allelic data of the 12 microsatellites (MS) analyzed were obtained from 78 of 146 (53.4%) parasite isolates in which 18 of 42 (42.8%) were pre-ACT and 60 of 104 were post-ACT. Additional allelic data of 10 MS were obtained from 43 of 146 (29.5%) parasite isolates, from which 11 of 42 (26.2%) were pre-ACT and 32 of 104 (30.7%) were post-ACT. Allelic data of parasites with 10 MS or more were included in the subsequent analysis where 121 of 146 (82.9%) parasites isolates were included; 29 of 42 (69.0%) were pre-ACT and 92 of 104 (88.5%) were post-ACT. Polyclonal, Multiple Infection (MI) parasite samples were determined by the number of alleles per locus; parasites with more than one allele at any of the loci were considered to have MI. For pre-ACT parasites, 19 out of 29 (65.5%) were polyclonal, whereas for post-ACT, 74 of 92 (80.4%) were polyclonal.

### Parasite genetic diversity

The 12 MS were highly polymorphic both in pre-ACT and post-ACT parasites. TA109 was most polymorphic with 35 alleles, whereas TA60 was the least polymorphic locus with 8 alleles. The diversity in pre-ACT parasite sample isolates was lower compared to post-ACT. The mean number of alleles in all the loci for the pre-ACT parasite samples was 13.2 (range = 8–18) compared to post-ACT which was 28.8 (range = 15–35); the post-ACT samples had significantly higher number of alleles in all the 12 MS loci compared to pre-ACT (Table 1; p < 0.0001).

**Table 1:** Genetic diversity of pre- and post- ACTs parasites at the 12 MS loci.

	pre-ACTs				post-ACTs			
	$\underline{\underline{n=\;29}}$				$\underline{\underline{n=\;92}}$			
LOCUS	CHR	N	Na	H_E_	N	Na	H_E_	F_ST_
**Polyα**	4	27	16	0.942	89	33	0.967	0.010
**TA42**	5	28	11	0.805	91	24	0.870	0.022
**TA81**	5	28	10	0.857	91	32	0.942	0.041
**TA1**	6	29	12	0.903	86	31	0.954	0.036
**TA87**	6	27	15	0.908	90	34	0.953	0.017
**TA109**	6	28	18	0.951	88	35	0.953	0.021
**TA40**	10	25	18	0.947	85	32	0.944	0.027
**2490**	10	28	13	0.883	88	24	0.922	0.018
**ARAII**	11	29	15	0.900	91	27	0.943	0.022
**PfPk2**	12	29	13	0.895	91	30	0.953	0.027
**PfG377**	12	29	9	0.806	88	15	0.734	0.073
**TA60**	13	26	8	0.830	83	28	0.935	0.052
**MEAN**		28	13	0.886	88	29	0.922	0.030
**SE**		0.372	0.960	0.015	0.763	1.629	0.019	0.005

Na = number of alleles for microsatellite loci, H_E_ = Expected heterozygosity, F_ST_- Fixation index statistics; Mann Whitney (p = 0.0226; 95% CI). Normalized microsatellite data was used to generate the above data using GenAlEx software.

Unbiased expected heterozygosity (H_E_) is a measure of the standardized genetic diversity factor in sample size of different populations. From the number of distinct alleles observed at each locus, the H_E_ was estimated per locus as shown in Table 1. The mean H_E_ for post-ACT parasite population was significantly higher than for pre-ACT parasite population (p = 0.0226). With the exception of 2 loci (TA40 and PfG377), all loci in the post-ACT parasite population had higher genetic diversity than the pre-ACT parasite population. However, most alleles were distributed widely across the two populations; the post-ACT parasite population had a large number of private alleles compared to pre-ACT with 19.8 and 4.2 private alleles respectively. The Shannon diversity index (I) is a quantification measure of abundance and evenness of species present, which gives more information on the composition of a population. The Shannon diversity index for pre- and post-ACT population was 2.3 and 3.0 respectively, further confirming higher genetic diversity in post-ACTs population.

### Parasite population structure

The analysis of molecular variance (AMOVA) revealed that most (95.7%) of the variance in allele frequencies was among individuals within the populations, whereas only 4.3% of the variation was explained by differences between pre- and post-ACT parasite populations. Wright’s F-statistics revealed that 8 of 12 microsatellite markers showed moderate genetic differentiation. The mean genetic differentiation index (F_ST_) among the pre-ACT and post-ACT parasite populations for all the 12 MS loci was 0.043, reaching statistical significance (p = 0.027, Table 2). The PfG377 locus had the largest F_ST_ (0.13) whereas Polyα had the lowest (0.007).

**Table 2:** F_ST_ values across the 12 MS loci for pre- and post-ACTs parasites.

Locus	Mean N	F_ST_
**Polyα**	116	0.0077
**TA42**	119	0.0302
**TA81**	119	0.0660†
**TA1**	115	0.0576†
**TA87**	117	0.0209
**TA109**	116	0.0292†
**TA40**	110	0.0400†
**2490**	116	0.023
**ARAII**	120	0.032†
**PfPk2**	120	0.0406†
**PfG377**	117	0.1285†
**TA60**	109	0.0852†
**Total Mean**	116.2	0.04287(SE .005)

N = total number of isolates, F_ST_ = Wrights F statistics,

^†^ indicates F_ST_ values with p < 0.05 (95% CI) which 8 loci out of 12 account for population structure seen between pre- and post-ACTs populations which do not share any genetic diversity.

Interpretation of the F_ST_ values at each locus was based on three categories defined earlier as no differentiation (0), low genetic differentiation (0–0.05), moderate differentiation (0.05–0.15) and great differentiation (0.15–0.25) [23,24].

Multi-locus genetic analysis of the 12 MS showed there were no matching haplotypes; a total of 121 (29 pre-ACT and 92 post-ACT parasites) unique haplotypes were generated. The highest multi-locus combination was seen in two samples pairs; one in the pre-ACT parasite population which had 8 of 12 of loci matching and the second pair in the post-ACT parasite populations in which 9 of 12 loci were matching.

**Fig 1:**
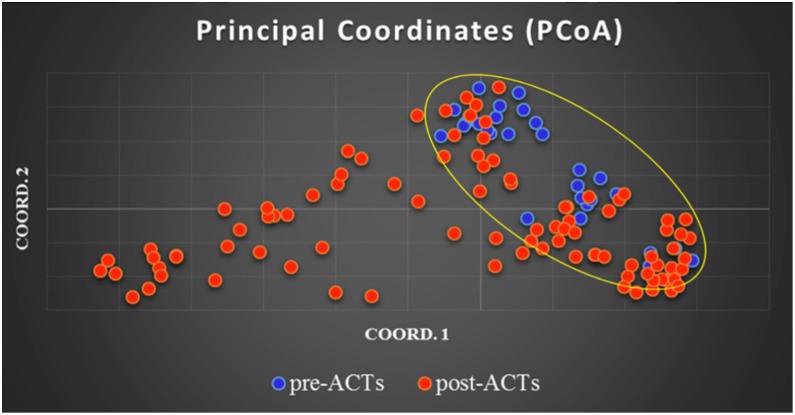
Principal Coordinate Analysis. Microsatellite data for both pre- and post-ACTs were used to find out patterns and relationships within a multivariate dataset. This graph was plotted using genetic distance matrix; blue dots for pre- and red dots for post-ACTs populations. This data shows the separation of the parasite genetic profiles in the two ACTs eras.

To perform the principle coordinate analysis, the eigenvalues and eigenvectors were calculated and the first two coordinates used to plot the genetic distance matrix (Fig 1). The percentage of variation explained by the two coordinates account for 8.9% and 5.4% respectively, revealing genetic difference in the two populations.

Analysis of multilocus index of association using the Monte Carlo method was performed (100,000 random re-sampling) to test the significance of LD both for the complete data set well as the individual populations. Significant LD was observed in the two populations’ samples from western Kenya for the complete data set. The post-ACT had the most significant index of association values (0.05; *p* < 0.00001) while pre-ACT had the least significant index of association values (0.019; *p* = 0.0036).

### Single Nucleotide Polymorphism Genotyping

Seventy-four parasites collected on day 0 when the patients were enrolled into the study and 18 recurrent samples were genotyped. The recurrent samples collected either on day 21, 28, 35 or 42 were determined to be re-infections. A total of 91 SNPs found across the *P*. *falciparum* genome were genotyped. Of the 91 SNPs genotyped, 78 (85.7%) produced robust genotype data which were analyzed. For each SNP, the number of parasite isolates that were successfully called is shown in S1 Table; the mean was 68.6 parasite isolates (range = 60–74) across all 78 SNPs. Parasites with < 5% of the 78 SNPs as mixed genotypes were considered to be single-clone infections. Of the 74 day 0 samples analyzed, 23 (30.6%) were single-clone infections, in which 15 (20.3%) had robust genotype data and were used in subsequent analyses. A multilocus combination analysis of the 78 SNPs showed no matching multilocus haplotypes. This generated a total of 15 haplotypes in which each sample showed a unique genotype combination. The highest multilocus combination was present in 9 isolates which matched in 19 out of 78 (24.4%) loci.

### Clearance slope half-life

The clearance half-life of parasite-infection of 74 post-ACT samples was obtained. Detailed information on this study will be reported elsewhere. The mean parasite clearance half-life was 2.63 hours (95% confidence interval [CI], 2.44–2.81) and the median clearance half-life was 2.55 hours (interquartile range [IQR], 2.02–3.09). The parasite clearance half-life ranged from 1.19–5.05 hours.

#### Effect of parasite genotype on parasite clearance half-life

**Fig 2:**
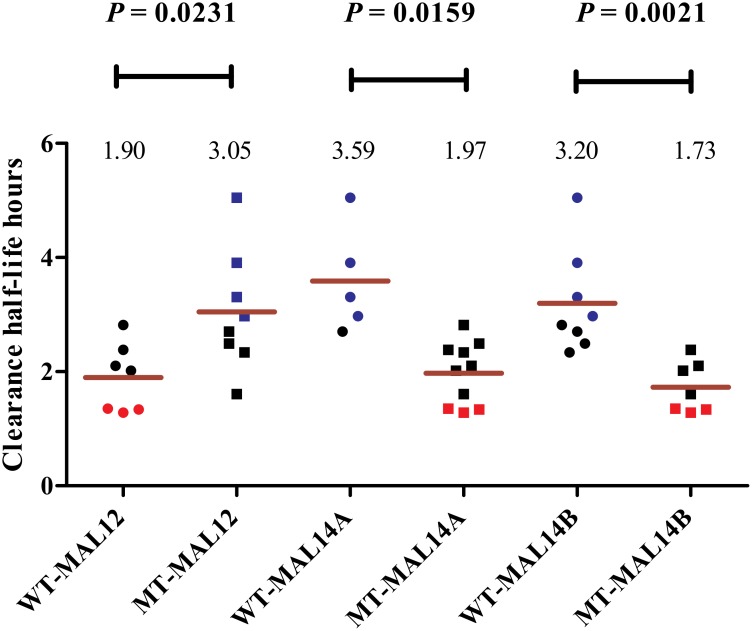
Correlation between 78 genome-wide SNPs and parasite clearance half-life. The 3 SNPs out of 78 genome-wide SNPs genotyped which showed positive correlation with clearance half-life with statistical significance (p < 0.05; CI 95%). The SNPs are as follows: *MAL12-1156125 (MAL12), MAL14-1199184 (MAL14A) and MAL14-3017684 (MAL14B) showing their wild type (WT) and mutant (MT) states.

To determine whether parasite genotype influenced parasite clearance rates, the half-lives of parasites carrying either a wild type or mutant allele at each SNP locus were compared. Non-parametric unpaired t-test (with Welch’s correction) revealed 3 SNPs in chromosome 12 and 14 (MAL12-1156125, MAL14-1199184, and MAL14-3017684) reached statistical significance (S2 Table). For the MAL12-1156125 locus, parasites carrying a mutant allele had slower clearance rates compared to wild type. Interestingly, however, for the MAL14-1199184 and MAL14-3017684 loci, parasites carrying the wild type alleles had slower clearance half-lives compared to ones carrying the mutant alleles (Fig 2).

Parasites with the same genotype at the 3 loci had similar clearance rate phenotypes; four of the parasite infections with the slowest clearance half-lives had the same genotype in which they carried a mutant allele at MAL12-1156125 and wild type alleles at MAL14-3017684 and MAL14-1199184 (Fig 2, shown in blue). Conversely, three of the parasite infections with the fastest clearance half-lives had the same genotype; they carried the wild type allele at MAL12-1156125 and mutant alleles at MAL14-1199184 and MAL14-3017684 (Fig 2, shown in red). Further, odds ratio analysis of the three SNPs (MAL12-1156125, MAL14-1199184 and MAL14-3017684) showed a positive association with the parasite clearance phenotype. In MAL12-1156125 SNP, the mutant allele was associated with delayed parasite clearance whereas in MAL14-1199184 and MAL14-3017684 SNPs, wild type alleles were associated with delayed parasite clearance (Table 3). Bonferroni's multiple comparison test showed significant correlation between MAL12-1156125 SNP mutant allele; MAL14-1199184 and MAL14-3017684 SNPs wild type alleles with delayed clearance rate as shown in Table 4 (p < 0.05%, 95% CI)

**Table 3:** *P*. *falciparum* SNPs significantly associated with delayed parasite clearance rates.

Chromosome	SNP (allele)	Allele frequency	OR (95% CI)	*P* value
12	MAL12-1156125 (T/**C**)	0.474	6.00 (1.33–27.06)	0.0322
14	MAL14-1199184 (**T**/A)	0.439	6.00 (1.22–29.46)	0.0324
14	MAL 14–3017684 (**A**/T)	0.438	9.75 (2.28–41.68)	0.0016

The Odds Ratio of infection having half-life > 2.55 hours is indicated with allele shown in bold compared to alternate allele. Sample size used for statistics in each locus is as follows: MAL12-1156125 (n = 35), MAL14-1199184 (n = 33), MAL 14–3017684 (n = 41).

**Table 4:** Post-test Analysis of the 3 SNPs using Bonferroni’s multiple comparison test.

Bonferroni's Multiple Comparison Test	Mean Diff.	t	Significant P < 0.05	95% CI of diff
WT-MAL12 vs MT-MAL12	-1.150	2.900	No	-2.390 to 0.08981
WT-MAL12 vs WT-MAL14A	-1.690	3.768	**Yes**	-3.093 to -0.2876
WT-MAL12 vs MT-MAL14A	-0.07484	0.1982	No	-1.255 to 1.106
WT-MAL12 vs WT-MAL14B	-1.301	3.281	**Yes**	-2.541 to -0.06119
WT-MAL12 vs MT-MAL14B	0.1726	0.4214	No	-1.108 to 1.453
MT-MAL12 vs WT-MAL14A	-0.5403	1.237	No	-1.906 to 0.8254
MT-MAL12 vs MT-MAL14A	1.075	2.959	No	-0.06114 to 2.212
MT-MAL12 vs WT-MAL14B	-0.1510	0.3942	No	-1.349 to 1.047
MT-MAL12 vs MT-MAL14B	1.323	3.336	**Yes**	0.08276 to 2.562
WT-MAL14A vs MT-MAL14A	1.616	3.850	**Yes**	0.3034 to 2.928
WT-MAL14A vs WT-MAL14B	0.3893	0.8914	No	-0.9764 to 1.755
WT-MAL14A vs MT-MAL14B	1.863	4.153	**Yes**	0.4602 to 3.266
MT-MAL14A vs WT-MAL14B	-1.226	3.374	**Yes**	-2.363 to -0.08986
MT-MAL14A vs MT-MAL14B	0.2474	0.6553	No	-0.9331 to 1.428
WT-MAL14B vs MT-MAL14B	1.474	3.716	**Yes**	0.2338 to 2.713

**Fig 3:**
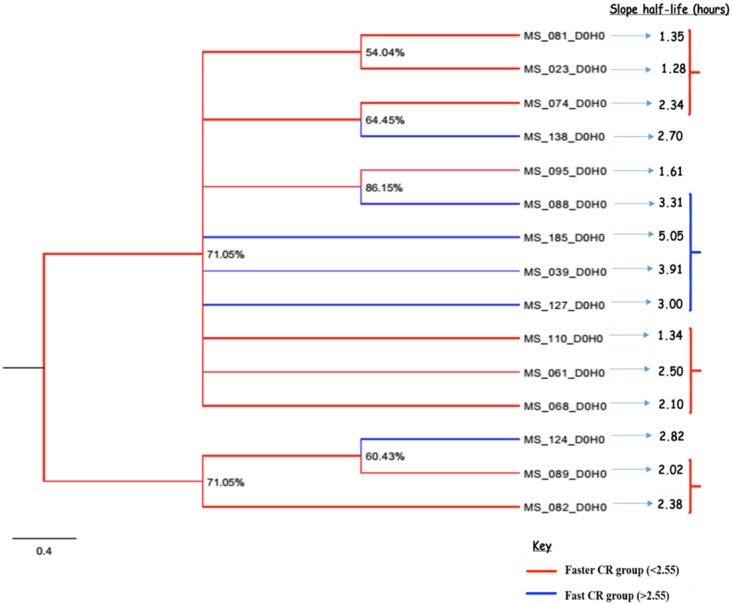
Bayesian midpoint tree showing single cloned *P*. *falciparum* haplotypes cluster. The cluster is in relation to clearance rates (slope half-life in hours). This was constructed using 78 concatenated SNPs whose genetic variants at each point were used to construct parasite relatedness using MrBayes software. A 10,000,000 generations was used to run and construct the tree which gave standard deviation below 0.01 to generate higher posterior probability values.

To evaluate the relatedness of parasite genotypes to parasite clearance half-life, the single-clone infections (based on the 78 SNPs) were analyzed using the Bayesian midpoint tree. Since all the parasites in this study can be categorized as fast-clearing (compared to SEA), parasites were stratified based on the median clearance half-life of 2.55 hours. Those above the median half-life were classified as fast clearing (Fig 3; shown in blue) and those below the median half-life as faster clearing (shown in red).

**Fig 4:**
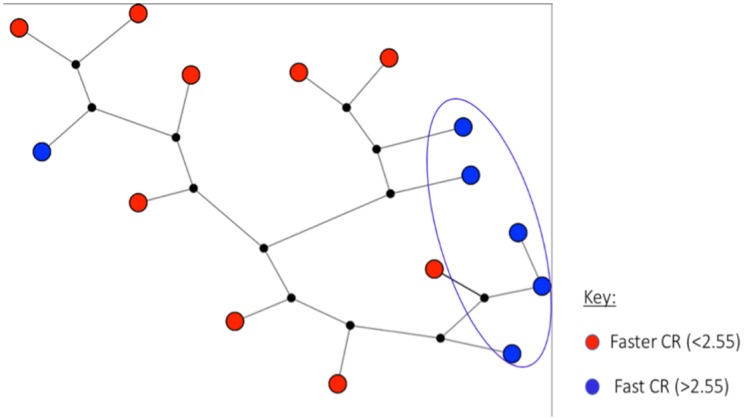
SNP-based genotypes and genetic variation seen in 15 single clones in Western Kenya in 2013–2014. Median-joining network diagram above shows genetic relationship of the western Kenya samples using 78 SNP haplotypes. Each circle in the network represents a unique haplotype profile with the size of the circle being proportional to the number of clones showing that particular haplotypes. The circle shown in red stands for samples with clearance rate < 2.55 slope half-life (hours) while those colored in blue represent those with clearance rate >2.55 hours. The black dots are hypothetical median vector generated by the software to connect existing haplotypes within the network with maximum parsimony.

Of the 15 single-clone infections, 6 were fast clearing and 9 were faster clearing. The median [range] age of the fast clearing subjects was 8.7 years [5–51.7] and for the faster clearing subjects was 6.0 years [1.4–24.3]. Of the 6 fast clearing parasites, 4 with the slowest clearance rates showed higher genetic relatedness by clustering together, whereas most of the faster clearing parasites showed higher genetic relatedness by clustering together. For the faster clearing parasites, two distinct groups were observed, each with at least 3 parasites in each cluster. Clustering of the samples of similar parasite clearance rate is also seen in Fig 4. The clustering does not seem to be impacted by patients’ age, although it is inconclusive to infer the importance of age as it relates to patient’s immunity given the small sample size.

## Discussion

Western Kenya is a malaria endemic region with stable, high transmission throughout the year with seasonal peaks [25–27]. Using MS profile analysis, previous studies have shown parasites in western Kenya have high genetic diversity with high infection complexity [28,29]. In line with previous studies, our data showed presence of high genetic diversity in western Kenyan parasites with post-ACT parasites showing significantly higher genetic diversity than pre-ACT parasites; the post-ACT parasites had higher number of alleles (including private alleles) in the 12 MS loci analyzed. None of the parasites analyzed had matching haplotypes. Further, genotyping of post-ACT parasites with half-lives at 78 loci distributed across the genome revealed 3 SNPs on chromosome 12 and 14 that might important in parasite clearance rates. This data is critical and requires additional studies to verify the findings. Most importantly however, we showed there are parasites from western Kenya with genetically determined responses to artemisinin treatment.

Our data clearly demonstrate that pre-ACT parasites had lower genetic diversity compared to post-ACT parasites. To offer an explanation to this finding, we considered the drug pressure exerted on these two parasite populations. Chloroquine was the treatment of choice for uncomplicated malaria infections in Kenya until 1998 [30] when Sulphadoxine-Pyrimethamine (SP) was introduced as the first-line treatment [31]. AL replaced SP as the first-line treatment in 2006 [32] and has been widely available in public and private clinics, and hospitals. Chloroquine and SP were replaced as first-line malarial treatments due to wide-spread treatment failure attributed to drug resistance [30,31,33]. In a series of studies conducted in Kisumu (which is located in western Kenya) in 1996, results showed that 85% of children under 5 years failed chloroquine treatment [30] and two years after SP was introduced as the first-line treatment, the estimated SP treatment failure rate was 42% [34]. This indicates that the pre-ACT parasite population in Kenya was under high drug pressure which might have forced the parasite to acquire genetic variants, including those that confer drug resistance resulting in reduction of parasite genetic diversity in the population.

Introduction of AL in 2006 coincided with scaling up of malaria control interventions which not only made the drugs readily available, but included other aspects of malaria control such as increased coverage of insecticide-treated net [31]. Parasite genetic variants are associated with a biological fitness cost which upon release of the pressure and with parasite outcrossing, leads to the loss of the acquired genetic variants in a population [15]. Change of drug policy in Kenya from SP to AL and the scaling up of other malaria intervention strategies released the high SP and chloroquine drug pressures on parasite populations, resulting in high genetic variability in post-ACT parasite population. Withdrawal of chloroquine and the introduction of AL has been shown to lead to progressive resurgence of chloroquine sensitive parasite populations [35,36,37,38,39]. AL remains highly efficacious in SSA and in Kenya [26,40,41] indicating the drug pressure has not yet selected for any genetic variants that would arise in parasites with attenuated or resistant phenotype. This can be interpreted to mean that selection of parasite populations by AL in post-ACT parasite is not as intense compared to pre-ACT selection caused by chloroquine and then SP. The strategy of combined therapy as opposed to mono therapy is important as well. Additionally, AL has been shown to select for chloroquine sensitive parasites [35,36,42,43,44,45], which diversity in these parasite populations [chloroquine sensitive] has been shown to be high [35,46].

Studies have shown Kenyan parasite populations have low genetic differentiation [28,47,48]. Similarly, our study revealed parasite populations had low genetic differentiation with the overall mean F_ST_ of 0.0427. The low to moderate differentiation observed between the pre- and post-ACT era can possibly influence population structure through selection and genetic drift. Most important, principle coordinate analysis revealed that parasites from the pre-ACT clustered tightly than the post-ACT. The parasites from the pre-ACT era depicted a panmictic population with less genetic distance between the parasites compared to the post-ACT parasites except for a few parasite populations that showed some structure.

Studies conducted in Zimbabwe and the DRC, regions of high malaria transmission described these parasite populations as having high genetic diversity and significant LD [16,49]. The non-random associations of alleles have significant implication for the spread of multi-locus drug resistance haplotypes with high levels of inbreeding, which increases their dispersal [50]. Within the pre- and post-ACT era parasite populations, a large proportion of multiple infections was found and therefore cross fertilization and recombination between distinct parasite genomes was expected to maintain the random association among the loci. The observed significant LD suggests the occurrence of inbreeding even in areas with intense and perennial malaria transmission which may explain the phenomenon of spread of drug resistance haplotypes across time. The data obtained in our study is consistent with the trends observed in Zimbabwe and the DRC.

In this study, we described three SNPs that might have some causal association with parasite clearance rates. These are nonsynonymous SNPs, one on chromosome 12 and two on chromosome 14. The SNP on chromosome 12, MAL12_1156125, is located in the gene PFL1375w and the SNPs on chromosome 14, MAL14_1199184 and MAL14_3017684, are found in genes PF14_0282 and PF14_0708 respectively (www.plasmodb.org). PFL1375w is a gene encoding a conserved *Plasmodium* protein with unknown function, PF14_0282 is a putative acid phosphatase, involved in malaria parasite metabolic pathways including drug metabolism. The phosphatase have been shown to interact with the proteins and enzymes involved in glycolysis pathway. These studies were conducted by genome wide in silico analysis of *P*. *falciparum* phosphatome [51]. PF14_0708 is a probable putative protein with unknown functions. However, since only a small number of samples were tested, additional studies are needed to determine if indeed there is any causal association between these SNPs and artemisinin sensitivity.

In low transmission setting such as SEA, patients are more often infected with CI parasites which arise due to low parasite genetic diversity and inbreeding [29,52]. Similar to identical twin studies in humans, CI parasites provide an effective approach to determining if phenotypic traits have a genetic basis [53]. Artemisinin resistance has been reported in SEA [2,3,54] with recent studies showing that the resistance phenotype can be explained by heritable genetic traits in the parasite population [14,52,55]. There are no reported cases of delayed parasite clearance in SSA, with studies reporting median half-lives of less than 3 hours [6, 9]. However, in the Kenyan coast, a decline of parasitological response rates to ACT treatment has been reported [56]. The authors in this study speculated it might be due to emergence of parasites with reduced drug sensitivity, reduction in population-level clinical immunity, or both. Similar to the previous findings, our study revealed that the median half-life was 2.55 hours, with a range of 1.19–5.05. Although western Kenya is a high transmission area with no reports of delayed parasite clearance to artemisinin treatment, Bayesian midpoint tree genetic analysis revealed parasites that clustered together based on parasite clearance were more genetically related.

High genetic variability in SSA parasite populations might be preventing emergence of parasites that are resistant to artemisinin. Antimalarial drug resistance may emerge *de novo* when malaria parasites with spontaneously arising mutations or gene duplications conferring reduced drug susceptibility are selected for by antimalarial drugs [57,58]. These emerging variations (mutations or gene duplication) might have relatively small phenotypic effects when acting individually. It is through the process of gene recombination that may occasionally produce a parasite possessing a ‘winning combination’ of alleles that cumulatively might confer resistance to antimalarial drugs, but must also provide a certain level of biological fitness for parasites to survive [15]. However, with high genetic diversity and outcrossing, the progeny can easily loose the ‘winning combination’. It is therefore likely that with proper management of combination therapies such as AL and monitoring of drug efficacy, artemisinin resistant ‘winning combination’ alleles might not easily emerge in high transmission regions such as western Kenya due to high parasite genetic diversity.

Although artemisinin resistant parasites have been reported in SEA, high grade resistance is yet to be reported [14]. However, genetically determined resistance to artemisinin is now prevalent in SEA and subpopulations associated with clinical resistance to artemisinin have been described [15]. The prevalence of K13 SNPs correlated with resistance among the Cambodian parasite subpopulations is associated with artemisinin resistance [10]. Since K13 SNPs have been shown not be prevalent or associated with artemisinin resistance in African parasites [11–13], identifying parasites with a genetically determined response to artemisinin resistance or identifying such subpopulations will be critical in identifying effective molecular markers for monitoring emergence and spread of artemisinin resistance in SSA. In this study, we have demonstrated the existence of parasites from western Kenya with a genetically determined response to artemisinin treatment. The analysis of K13 SNPs in samples from this study will be reported elsewhere.

Our study had several limitations. First, the sample size was small since it is difficult to obtain single-clone infection in high transmission areas. Second, the parasites had a median half-life of 2.55 hours and can therefore be considered fast clearing. Third, none of the single-clone parasites were CI. Regardless of the study limitations, our study show that parasite genetics might account for the variation in clearance rates. However, there are many factors that confound clearance rates [59]. Efficacy studies with large sample size and additional analyses are required to further elucidate these findings. Further, it will be important to conduct studies in areas with different transmission intensities including low transmission areas in Kenya where malaria endemicity is similar to that found in SEA. This will be crucial because clinical immunity at a population-level is a critical confounding factor in determining response to ACTs treatment and parasite clearance rates [7,60].

In conclusion, we have showed for the first time the existence of parasites with genetically determined responses to artemisinin treatment in SSA. It is expected the proportion of variation in parasite clearance attributable to parasite genetics will increase at a much slower rate in high transmission areas of SSA than it is in SEA where transmission is low. However, there are some areas in SSA that have experienced a reduction in malaria transmission or are low transmission areas [61,62] with more structured parasite populations [63], and are therefore more likely to develop parasites with artemisinin resistant ‘winning combination’. As such, surveillance of artemisinin resistant parasites should not only focus in high transmission areas in SSA but also in regions of low transmission.

## Materials and Methods

### Ethics Statement

Both studies were reviewed and approved by the Ethical Review Committee of the Kenya Medical Research Institute (KEMRI), Nairobi, Kenya and Walter Reed Army Institute of Research (WRAIR) Institutional Review Board, Silver Spring, MD. The pre-ACT study and post-ACT studies were conducted under approved study protocols numbers: KEMRI-SSC 1330/WRAIR 1384 and KEMRI-SSC 2518/WRAIR 1935 respectively. Patients presenting with uncomplicated malaria at the study site were enrolled into the study after seeking informed consent per the study protocol. All potential study subjects provided written informed consent before screening, enrollment and had to pass an assessment of understanding. Protocols used in these studies complied with International Conference on Harmonization Good Clinical Practice (ICH-GCP) guidelines. These studies were conducted in accordance with the Declaration of Helsinki and the Belmont Report including all federal regulations regarding the protection of human participants as described in 32 CFR 219 (The Common Rule). Internal policies for human subject protections and the standards for the responsible conduct of research of the US Army Medical Research and Materiel Command were also followed. WRAIR holds a Federal Wide Assurance from the Office of Human Research Protections under the Department of Health and Human Services. Accordingly, all key study personnel in both studies were certified as having completed mandatory human research ethics education curricula and training under the direction of the WRAIR IRB Human Subjects Protection Program.

### Parasite isolates, eligibility criteria and study site

The pre-ACT isolates (n = 29) used in this study were from cryopreserved archived samples collected in Kisumu County, western Kenya between 1995 and 2003. This was before AL was adopted as the first-line treatment for uncomplicated malaria in Kenya. The post-ACT samples (n = 75) were from an in vivo efficacy study conducted in 2013–2014 in Kombewa district hospital in Kisumu County, western Kenya; eight years after AL was rolled-out as the first-line treatment for uncomplicated malaria. On enrollment, the patients’ demographics, (including age and gender), place of birth, place of residence, occupation and travel history in the last 2 months was captured on a clinical data sheet in both studies. Patients presenting with uncomplicated malaria, aged between 6 months and 65 years were consented. Eligible criteria included: measured temperature of ≥ 37.5°C, or history of fever within 24 hours prior to presentation and mono-infection with *P*. *falciparum*. In addition, the study subjects had a baseline parasitemia of 200–200,000 asexual parasites/μl. The presence of malaria was confirmed by microscopy and rapid diagnostic test (RDT; Parascreen^®^, Zephyr Biomedicals, Verna Goa, India). Whole blood was collected and aliquots stored for future analysis were cryopreserved in cryovials in liquid nitrogen tanks as specified in the study protocols.

The study subjects were treated with oral AL (Coartem) administered over three consecutive days, a standard of care for *P*. *falciparum* malaria in Kenya. Those who met the eligibility criteria were referred to the clinic. Additionally, for those who were enrolled in the efficacy trial (KEMRI-SSC/WRAIR 1935), they were admitted at the study facility for approximately 3 days. A written informed consent was provided by study participants and/or their legal guardians. Parasite clearance was monitored microscopically after every 6 hours. Whole blood was collected and aliquots preserved for analysis as specified in the study protocols.

### Parasite clearance rate calculations

Parasite clearance rates were calculated using the Worldwide Antimalarial Resistance Network (WWARN) tool for Parasite Clearance Estimator (PCE) located at http://www.wwarn.org/toolkit/data-management/parasite-clearance-estimator. Log transformed parasite densities were plotted against time in hours to generate the slope half-life which is defined as the time needed for parasitemia to be reduced by half. This constant is independent of the starting value of parasitemia. The half-lives were calculated as follows:

T 12=loge (2)/k=0.692 k,

where k is the clearance rate constant

### *P*. *falciparum* DNA extraction and parasite SNP genotyping

Genomic DNA was extracted from whole blood using QIAamp Blood mini kit (Qiagen, Valencia, CA, USA) as recommended by the manufacturer. Extracted DNA was stored appropriately until analyzed. Ninety one single nucleotide polymorphisms (SNPs) distributed across all the 14 chromosomes of *P*. *falciparum* genome previously described by Phyo et al. [14] were analyzed using PCR-based single-base extension on Sequenom MassARRAY platform (Agena Biosciences, San Diego, CA, USA) following manufacturer recommendations. The PCR and extension primers used in the analysis of the 91 SNPs can be found in the S3 Table while flanking sequences used to design multiplex assays are found in S4 Table.

Single clones were isolates that showed < 5% of the total SNPs genotyped.

### Genotyping of microsatellite loci

Parasites were genotyped using previously described twelve polymorphic microsatellite markers which are distributed across the genome [16]. These include Polyᾳ (Chromosome 4), TA42 (Chromosome 5), TA81 (Chromosome 5), TA1 (Chromosome 6), TA109 (Chromosome 6), TA87 (Chromosome 6), TA 40 (Chromosome 10), 2490 (Chromosome 10), ARAII (Chromosome 11), PfG377 (Chromosome 12), PfPk2 (Chromosome 12), and TA60 (Chromosome 13). Reference clone 3D7 was used as a control. PCR reaction and analyses were performed as previously described [16,64].

Length variation of labeled PCR products was measured on an ABI 3130/3500xL Genetic analyzer (Applied Biosystems, Foster City, CA, USA) by running the samples alongside the GS500LIZ size standard (Applied Biosystems, Foster City, CA, USA) as recommended by the manufacturer. Estimates of DNA fragments size relative to the size standard (LIZ500) were generated and calculated in allele length and the peak heights quantified (calculated in Relative Fluorescence Units—RFU).

#### Scoring of microsatellite allelic length variation and assessment of multiple infections

Multiple infections were defined as those in which one or more of the twelve loci showed multiple alleles. GeneMapper v 4.0/4.1 (Applied Biosystems, Foster City, CA, USA) was used to automate measurements of allele length and to quantify peak heights. Multiple alleles per locus were scored if electrophoretic peaks corresponding to minor alleles were ≥ 33% the height of the predominant allele in the isolate [16]. Allele scoring was determined for each parasite clone where the largest among all the alleles was considered the multiplicity of infection of that sample.

#### Estimation of genetic diversity for 1995–2003 and 2013–2014 parasite populations

Genetic diversity was measured by the number of alleles per locus and expected heterozygosity on the basis of allele frequency data in each parasite population. In the cases of presence of multiple alleles at a locus within an infection, only the predominant allele defined by the allele with the highest peak height in electropherograms was used for allele frequency calculations. This procedure is appropriate for estimation of population allele frequency if the composition of PCR products is representative of the composition of templates. The unbiased expected heterozygosity, H_E_ was calculated as:

$$H_{E}=\left[n/\left(n-1\right)\right]\left[1-\sum pi^{2}\right]$$

Where n is the number of isolates genotyped and pi is the frequency of the *i*^*th*^ allele. This was estimated using GenAlEx v2.2 [64] and confirmed using GENEPOP software [65]. The levels of diversity in different populations were compare using unpaired t-test and estimated effective population size using observed heterozygosity and number of effective alleles (Ne). Population subdivision was estimated using Wright’s *F* statistics.

#### Construction of Multi-locus Microsatellite haplotypes and Linkage Disequilibrium for 1995–2003 and 2013–2014 parasite populations

The predominant alleles at each locus were used to construct the infection haplotypes. We used a permutation procedure to test the null hypothesis of random association among loci for each parasite population [66,67]. The program LIAN, version 3 [68] was used to compute the number of alleles shared (D) between all pair wise comparisons of complete 12-locus haplotypes and to measure the variance of this distance measure (VD). To investigate if the observed data differed from random expectations, we compared the observed VD with the distribution of VD values in 100,000 simulated data sets in which alleles at each locus were randomly reshuffled among genotypes. We used the index of association (*I*_*A*_) to measure the strength of LD. A standardized *I*_*A*_ statistic (*I*_*A*_*s*), calculated as ((V_D_/V_e_ − 1)/(1 − r)), was used where r is the number of loci, a measure of haplotype-wide linkage and the 95% confidence limits determined by Monte Carlo simulations (LMC) [69].

#### Measurement of parasite relatedness

Relatedness is generally estimated for diploid species [70]. However, we measured the relatedness in blood-stage parasites between gametes of the single clones by the 78 assayed SNPs were analyzed by carrying out multiple sequence alignment using Muscle 5.8; the phylogenetic tree was built using MrBayes [71] and viewed using Figtree software [72]. Parasites that differed at less than 5%of loci were assumed to be identical-by-descent (IBD) and were referred to as being clonally identical (CI). We assessed the correlation between genetic variability and clearance rates as a phenotype to determine whether clones with similar phenotypic characteristics harbor similar genotypic features. We constructed median-joining networks in NETWORK version 4.6.1.1 (http://www.fluxus-engineering.com/sharenet.htm) to visualize the relationships among *P*. *falciparum* isolates from Pre- and Post- ACT era.

### Statistical analysis

Genotype calls were made using Mass Array SpectroTyper 4.0 software (Sequenom) that interprets the change in mass and converts to the nucleotide or SNP variants. To investigate the influence of genetics on clearance rates, correlation of the two parameters was carried out using GraphPad prism (GraphPad Software, Inc., San Diego, CA, USA).

To determine parasite genetic diversity and population structure, 12 MS were used to calculate allele frequencies, heterozygosity values, molecular variance, haplotypes, principal coordinates and fixation indices using GenAlEx and Arlequin software. Predominant alleles were used in calculation of allele frequencies. For H_E_ analysis, samples were grouped based on ACTs eras (pre- and post- ACTs). For this comparison, the mean H_E_ values were compared by using a Mann-Whitney U statistics implemented in the statistical package GraphPad Prism (San Diego, CA, USA). A p value of ≤ 0.05 was considered statistically significant.

## Supporting Information

S1 Table Call rate per SNP per every isolate genotyped. (XLSX)

S2 Table Mutant and wild-type genotype analysis of 78 SNPs for statistical significance. (DOCX)

S3 Table PCR and Extension Primer sequences for 91 SNPs found across *P*. *falciparum* genome. (XLSX)

S4 Table Flanking sequences for 91 SNPs for designing MassARRAY multiplex assays. (XLSX)
