# Supplementary material for: Genetically Determined Response to Artemisinin Treatment in Western Kenyan Plasmodium falciparum Parasites
Source: PLoS One. 2016 Sep 9;11(9):e0162524. doi: 10.1371/journal.pone.0162524 (PMC5017781; doi:10.1371/journal.pone.0162524)
Supplement: S2 Table — (DOCX) [file pone.0162524.s002.docx]

**S2 Table:** Genotype Analysis: H_0_ we hypothesized that there is no difference in clearance half-life in isolates with the SNPs (mutant) against those without the SNP (wild-type). P< 0.05 you reject the hypothesis above as you will be confident that the hypothesis above is not true.

| **SNP** | **P VALUE** | **SNP** | **P VALUE** |
| --- | --- | --- | --- |
| **MAL14-3017684** | **0.0006** | *MAL12-1591944 | <3 Values |
| *MAL05-385372 | 0.2149 | MAL04-469608 | 0.845 |
| MAL08-1292195 | 0.8639 | MAL08-1057901 | 0.5395 |
| *MAL04-268436 | 0.7182 | *MAL13-1233419 | 0.4396 |
| MAL12-2046727 | 0.8639 | MAL04-307321 | 0.8286 |
| MAL07-759749 | 0.6477 | MAL12-1131597 | 0.9546 |
| *MAL08-502163 | <3 Values | MAL07-476303 | 1.0000 |
| *MAL02-660994 | 0.8286 | MAL03-748204 | 0.3481 |
| MAL06-1206499 | 0.8591 | MAL13-146947 | 0.1447 |
| MAL07-1323949 | 0.3884 | MAL13-1966774 | 0.9551 |
| *MAL07-543849 | 0.3969 | MAL11-1802198 | <3 Values |
| MAL10-336274 | <3 Values | MAL11-1749804 | 0.2238 |
| MAL06-410691 | 0.3710 | *MAL12-83177 | 0.5941 |
| *MAL13-210569 | 0.1292 | *MAL12-529412 | 0.1645 |
| *MAL09-492618 | 0.2238 | MAL11-185094 | 0.5941 |
| MAL06-1184507 | 0.9451 | *MAL06-593821 | 0.8286 |
| *MAL11-281726 | 0.5135 | *MAL01-354427 | <3 Values |
| MAL01-539149 | 0.6787 | MAL14-1853537 | 0.1520 |
| *MAL04-809686 | 0.8591 | MAL04-994352 | 0.7756 |
| *MAL07-1311707 | <3 Values | MAL07-1122023 | 0.8286 |
| ***MAL12-1156125** | **0.0205** | MAL14-1458321 | 0.7679 |
| *MAL04-933828 | <3 values | MAL07-511352 | 0.4396 |
| MAL09-692487 | <3 values mutant column | MAL04-1140617 | <3 values mutant column |
| **MAL14-1199184** | **0.0013** | MAL03-439621 | 0.4363 |
| MAL04-393114 | <3 values mutant column | MAL08-477431 | 0.9480 |
| MAL10-1300115 | 0.1000 | MAL14-3124926 | 0.9425 |
| *MAL07-672051 | 0.3961 | MAL10-317580 | 0.2065 |
| MAL02-322398 | 1.0000 | MAL08-1101130 | 0.5135 |
| *MAL09-1111191 | 0.9480 | *MAL12-114173 | 0.9551 |
| MAL04-1101563 | 0.7441 | MAL11-769993 | <3 values mutant column |
